# A qualitative assessment of health seeking practices among and provision practices for men who have sex with men in Malawi

**DOI:** 10.1186/1472-698X-14-20

**Published:** 2014-06-03

**Authors:** Andrea L Wirtz, Dunker Kamba, Vincent Jumbe, Gift Trapence, Rehana Gubin, Eric Umar, Susanne K Strömdahl, Chris Beyrer, Stefan D Baral

**Affiliations:** 1 Center for Public Health and Human Rights, Department of Epidemiology, Johns Hopkins Bloomberg School of Public Health, 615 N. Wolfe St., Room E7144, Baltimore, MD 21205, USA; 2 Johns Hopkins Medical Institute, Baltimore, USA; 3 Center for the Development of People, Blantyre, Malawi; 4 Malawi College of Medicine, Blantyre, Malawi; 5 Centre for Global Health, Trinity College, Dublin, Ireland; 6 Jhpiego, Washington, DC, USA

**Keywords:** Health knowledge, Attitudes, Practice, HIV/AIDS, Men who have sex with men (MSM), Stigma, Malawi

## Abstract

**Background:**

In the context of a generalized epidemic and criminalization of homosexuality, men who have sex with men (MSM) in Malawi have a disproportionate burden of HIV compared to other adults. Past research has documented low uptake of HIV prevention and health services among MSM, self-reported fear of seeking health services, and concerns of disclosure of sexual orientation and discrimination in health settings. Qualitative research was conducted among MSM and health service providers in Blantyre, Malawi to understand underlying factors related to disclosure and health seeking behaviors and inform the development of a community-based comprehensive HIV prevention intervention.

**Methods:**

Using peer recruitment, eight MSM participants representing a range of ages, orientations, and social and behavioral characteristics were enrolled for in-depth interviews. Five service providers were recruited from the district hospital, local health and STI clinics, and a HIV prevention service organization. We use the Health Belief Model as a framework to interpret the influential factors on 1) health seeking and uptake among MSM, and 2) influences on provision of services by healthcare providers for MSM.

**Results:**

Results highlight disclosure fears among MSM and, among providers, a lack of awareness and self-efficacy to provide care in the face of limited information and political support. Service providers reported concerns of adverse repercussions related to the provision of services to men in same sex sexual relationships. Some MSM demonstrated awareness of HIV risk but believed that within the wider MSM community, there was a general lack of HIV information for MSM, low awareness of appropriate prevention, and low perception of risks related to HIV infection.

**Conclusions:**

Qualitative research highlights the need for appropriate information on both HIV risks and acceptable, effective HIV prevention options for MSM. Information and educational opportunities should be available to the wider MSM community and the health sector. Health sector interventions may serve to increase cultural and clinical competency to address health problems experienced by MSM. To ensure availability and use of services in light of the criminalization and stigmatization of same sex practices, there is need to increase the safety of uptake and provision of these services for MSM.

## Background

There is a solid foundation of data highlighting the high prevalence of HIV among MSM and the importance of this population to understanding the dynamics of all HIV epidemics, including generalized ones [1,2]. Risks for acquisition and transmission include biological risks of HIV transmission during anal sex, inconsistent condom use, genital sexually transmitted infections (STI), and mental health, which has been shown to be associated with elevated HIV risk status mediated through behavior [3-12]. The current content and coverage of HIV prevention programs, however, is insufficient in addressing HIV epidemics among MSM [13]. Coverage of existing interventions reaches less than 10% of MSM globally with far lower coverage in most low and middle income countries [14,15]. A range of biomedical and behavioral interventions [9,13,16] and comprehensive approaches have been proposed for HIV prevention for MSM [17]. Yet, linkages to these interventions for these men are necessary and often rely on positive patient-provider relationships for discussion of high risk behaviors, health outcomes, and intervention or treatment options. These discussions allow providers to assess the range of behaviors and health risks, including and beyond HIV, to provide comprehensive and specialized mental and physical health care to MSM [18].

While health service utilization by MSM and discussion of high risk practices with providers are fundamental to learning of and accessing HIV and STI prevention and treatment options, previous epidemiologic investigations of HIV and sociobehavioral risk factors among MSM in Malawi, Namibia, and Botswana found that only 9% of MSM in Malawi had ever disclosed sexual practices to a healthcare provider - 17% among the three Southern African countries studied [19,20]. Almost 20% of Malawian MSM reported ever being afraid to seek health services [20]. Another study conducted in central and southern Malawi similarly found that only 18% of MSM participants had ever been exposed to HIV prevention messages for MSM, and 31% feared disclosure of sexual orientation and discrimination [21]. Research among MSM in other sub-Saharan African countries have documented experienced and perceived sigma in healthcare settings [22-26], low prevalence of HIV testing [27-30], and low access to care for those who are living with HIV [31]. Yet, research from Kenya suggests that health care providers often lack professional training on specific health needs of MSM and appropriate risk reduction counseling, leaving them inadequately equipped to provide these needed services [32]. Given the importance of voluntary disclosure to health practitioners on the overall health and access to HIV prevention and treatment services for gay men and other MSM [18], these figures highlight the importance of understanding the gaps and needs for future HIV prevention among MSM, particularly for a population among whom the estimated HIV prevalence is 15 to 20% [19,33]. Though no current population size estimate has been published for MSM in Malawi, earlier estimates from Eastern and Southern Africa of suggested a range of 1-4% of men report male-male intercourse in the last year [34]. These estimates may be higher with the recent proliferation of research among MSM in Africa and across the globe. A focus on specifically addressing HIV risks and related health outcomes that are faced by men who practice same sex sexual relationships in Malawi is warranted, but highly challenged in Malawi, where homosexuality is considered an ‘unnatural offense’ and punishable with imprisonment [35]. Prior to 2012 (and at the time of this research), the National HIV Strategy for Malawi indicated a need to provide services for MSM, but did not offer clear guidance on how this was to be implemented [36].

The Health Belief Model, which posits that the likelihood of adopting a recommended preventive health action, is influenced by individual perceptions, modifying factors, cues to action and self-efficacy [37]. This explanatory theory focuses on the mechanisms that act on the individual level to influence health behavior. The theory has predominantly been used to promote positive behavior change among adolescents or evaluate a HIV intervention among adolescents in several Sub-Saharan countries, including Cameroon [38,39], Uganda [40], South Africa [41,42], Madagascar [43], and Guinea [44]. Specifically among MSM, the Health Belief Model has been predominantly utilized in the United States [45,46], including Puerto Rico [47], to promote sexual behavior change and condom use. These Health Belief Model -informed interventions were included among the behavior change studies and randomized clinical trials (RCTs) reviewed in a meta-analysis which indicated significant impacts in condom use during anal intercourse and reduction in numbers of sex partners [48]. While the model has also informed the basis of methamphetamine reduction interventions [49] and has been used to understand determinants of hepatitis B vaccine behaviors among MSM [50,51], our review of peer-reviewed literature did not uncover the use of Health Belief Model to evaluate or inform any sub-Saharan interventions to reduce behavioral risks for HIV infection among MSM. While many behavior change interventions and evaluations have been and are being conducted in Sub-Saharan Africa, they are not always identified as having one theoretical basis, though they may incorporate elements of one or many cognitive theories [52].

We used the Health Belief Model to understand and describe the dynamics of perceptions, modifying factors, and cues to action and the effect these have on health seeking practices and disclosure of sexual practices by MSM in Malawi. Access to and utilization of services by MSM and discussion of high risk sexual practices to inform healthcare decisions requires input by both the patient and service provider alike [18]; we therefore propose the use of the Health Belief Model to understand the perceptions and modifying factors that influence the preventive actions by health providers to discuss high risk practices and providing services to MSM. This research was conducted to inform the development of a combination HIV prevention intervention (CHPI) for MSM in Malawi.

## Methods

From May to July 2011, we conducted in-depth interviews among purposively sampled MSM and health service providers working in Blantyre, Malawi. Using purposive sampling and peer and key informant recruitment, a total of eight in-depth interviews were conducted with men who reporting sexual contact with another man in the last 12 months. These men represented a range of ages, social and behavioral characteristics, self-reported sexual orientations (gay, bisexual, heterosexual), and marital patterns (with a woman). Eligibility criteria for enrollment of MSM in the in-depth interviews also included being born male, aged 18 years or older, living in Blantyre for at least one year, and providing informed verbal consent to participate.

Health service providers were purposively sampled with an attempt to recruit staff from the local hospital, health clinics, and HIV prevention organizations. Eligibility criteria for these interviews included being aged 18 years or older, having worked in the specific health setting for one year or more, and providing informed verbal consent to participate. A total of five participants were recruited from the district hospital, local public health clinics, STI research clinics, as well as from an HIV prevention service organization. Service providers from public hospitals and clinics were selected for participation, recognizing that a large proportion of the adult population in Blantyre, including MSM, use public services. Their input would be important to understand the experiences of service providers and what interventions may be implemented to enable them to provide quality care to MSM.

Interviews among MSM and health professionals were conducted by local interviewers, with prior experience in qualitative research. Interviewers received additional training on confidentiality, human subjects research, and qualitative methods. Interviews were conducted in a private room at the study site, though health professionals could elect to be interviewed in their office or other preferred, private location. To further ensure privacy and confidentiality the study staff scheduled participants in such a way that participants did not overlap and unintentionally meet at the study premises. Participants also entered and exited the study office from different locations to minimized chances of interaction among individuals who may not have publicly disclosed their sexual preferences. In-depth interviews were guided by the use of semi-structured, translated interview guides. Interview questions and probes for the interviews with MSM were developed to assess perspectives on sociobehavioral and structural risks for HIV infection, experiences of stigma and discrimination related to same sex practices or HIV status, health service and HIV prevention utilization, and specific barriers or facilitators to accessing or providing care. Interview questions and probes for health professionals were developed to assess services provided to the general public and those provided (or not) to MSM; opinions of services and prevention programs that should be provided to these men; knowledge and perspectives on health and HIV risks among MSM; perceptions of stigma and discrimination towards MSM that may exist in the health sector; and barriers or facilitators related to providing care to MSM.

Interviews were conducted in the local language, Chichewa, and lasted between 30 to 60 minutes. The interviews were recorded, with the participants’ permission, transcribed, and translated for analysis. At the end of each day, the audio recordings were transferred to a password protected computer and removed from the digital recorders. Transcripts were independently analyzed by two investigators (AW, RG) using Atlas.ti (Cincom Systems, Berlin) and through application of grounded theory method to identify emerging themes and decide appropriate codes. Coded transcripts were then compared to resolve discrepancies between the two analysts. Secondary analysis was conducted to map these themes to the Health Belief Model to explain how perceptions and modifying factors impact the likelihood of health seeking behavior among MSM and impact service provision by health providers in Blantyre. Findings from these two populations are summarized below with key quotations included to contextualize the findings.

This study was reviewed and approved by the Johns Hopkins Bloomberg Institutional Review Board and the College of Medicine Review and Ethics Committee at the Malawi College of Medicine. In concordance with recommendations from the ethics committee to protect the privacy of participants, focus group discussions were not conducted. No personal identifiers of participants were collected at any point during the data collection. The use of verbal informed consents were approved by both ethical committees and used to further refrain from the collection of names or identifiers. The informed consent process included discussion of the study purpose, activities, benefits and risks to participation, dissemination and reporting of results, and study contact information. Results are presented as experiences and opinions of health professionals and MSM; we do not disclose the health or service organization that employed the health providers to further protect their anonymity.

## Results

**Figure 1:**
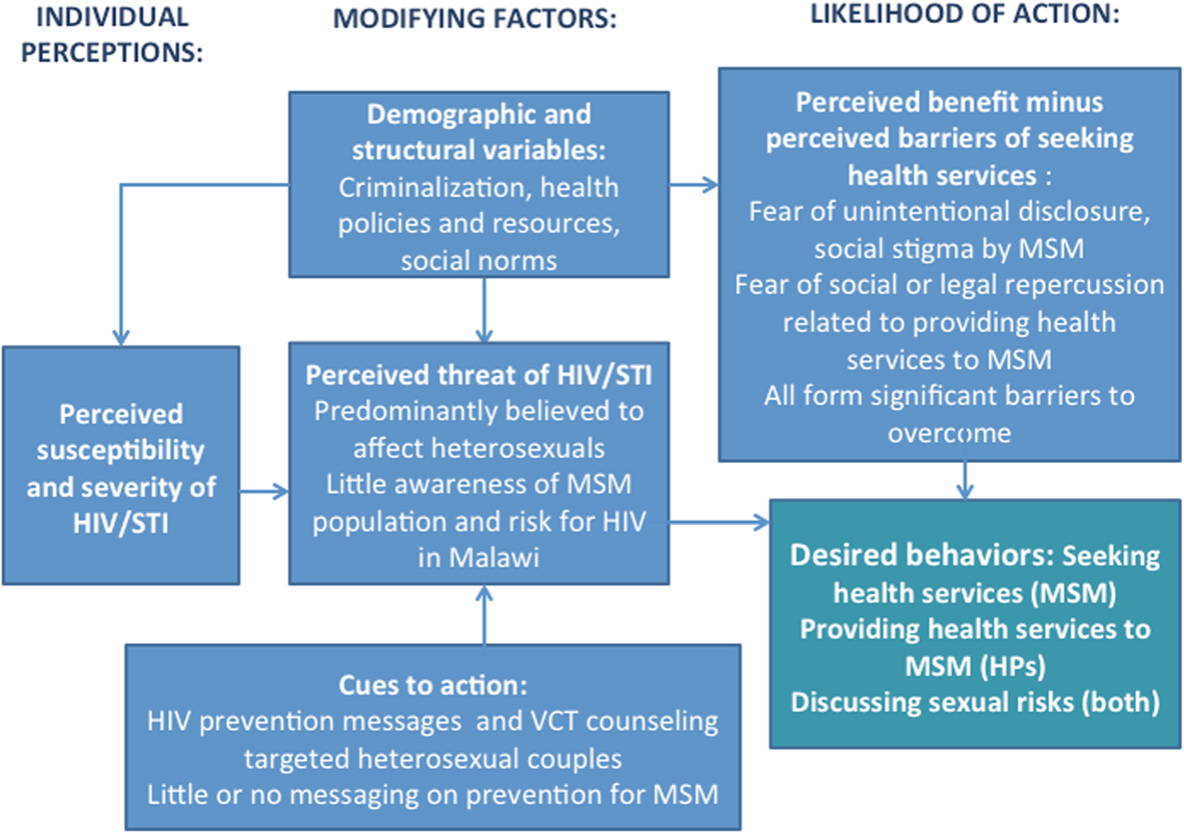
The Health Belief Model as applied to HIV and health service provision and uptake among men who have sex with men in Malawi.

Perceptions of risk, barriers, and benefits related to disclosure of same sex practices and health seeking behaviors, reported by health professionals and MSM alike, were salient themes uncovered during the primary analysis. Given these findings, we proceeded with the Health Belief Model to describe the dynamics of perceptions and other factors on individual health seeking, practices of health provision, and discussion of high risk sexual practices between provider and MSM in Malawi. Figure 1 overlays these perceptions among MSM and health providers against the Health Belief Model, depicting the modifying factors on the desired behavioral outcomes and providing context-specific examples of the factors identified by the research. The following quotes contextualize this description and are presented according to key domains of the Health Belief Model, though some quotations may be relevant across several domains.

### Individual perceptions: susceptibility, severity, and threat of HIV or STI infection

Perceived susceptibility and severity are individual subjective assessments of risk of developing a health problem and the potential severity and consequences of the health problem [37]. In this case, we focus on the perceptions of MSM and health providers in regards to risk of sexual transmission or acquisition of HIV or STI infection among MSM. Among participants this awareness of risk and perceived severity of these infections varied; some men reported that they were not aware of the risk for HIV acquisition and transmission associated with anal sex, and some believed that their risk for HIV infection was only possible, or was elevated, during sexual intercourse with a woman. The few who were aware learned about such risk through participation in a research study that was targeted to MSM. Perception of severity related to STIs was low as some men felt these infections could resolve on their own and proceeded without treatment (quotation included in next section).

…it’s like you want to meet your wife and you don’t know how your wife behaves - is she meeting other men? That is a very difficult thing. You can get infected with a certain disease if the wife has contracted a certain disease. She can, as well, infect you. - *Male Participant ID08*

…a lot of people do not believe that when you have sex with your fellow man you can contract infections. They think that amongst men you cannot infect one another. They think that you can get infection only if you have sex with a woman- that is when you can contract infections. It’s just a few people who are in the know that if you have sex with your fellow man you can infect one another… - *Male participant ID07*

Additionally, some health providers acknowledged the risk for HIV and STI among MSM and suggested improving risk awareness as a critical first step to HIV prevention among MSM.

I would say the first, the major issue for the MSM is awareness in terms of how dangerous it is, how they are more at risk when they practice anal sex, as opposed to other people. How important it is to protect themselves using condoms and using lubricants. - *Health Professional ID04*

Several providers were themselves unaware of the risk for HIV among MSM, though reported lack of awareness among their colleagues. This was reportedly influenced by public health policies and lack of information and messaging about risk for HIV among MSM. Without this information and without political support for providers to serve MSM, there was less perceived threat of HIV among MSM by the public and providers and, thus, less encouragement towards health seeking practices. One upper-level health provider suggested that information about population size and the epidemiology of HIV among MSM was needed to provide an evidence base that could be used to improve awareness among providers.

Now, you will find that the first instances of the meeting [with District and Health officers to talk about HIV among MSM] there is a lot of questioning. You can actually see from the faces, these people are really looking at us and are saying "WHAT?" What are these guys talking about? You know, we even get statements ‘does it [HIV among MSM] exist here’? Do you have any research to show that it exists here? So they are quite not aware and they doubt … You know there is already a resistance in the meeting just talking about men who have sex with men. - *Health Professional ID04*

### Modifying factors

Modifying factors in the Health Belief Model typically include a range of demographic, psychosocial, and structural factors that influence individual perceptions of health behaviors [37]. Key modifying factors in the course of health seeking behavior and access among MSM included criminalization of homosexuality, social stigma, limited health resources, and cues to action (see next Results section). These factors influenced perceptions of physical and social barriers among MSM. Furthermore, individual uncertainty of how one would be treated individually by staff and concerns that the service provider would report the same sex practice, served as a significant barriers to health seeking behavior even when MSM knew they needed to seek health services.

I have [known] individuals who have had sexual transmitted infections. For some time they may have it. They don’t seek treatment and they just walk around with it. Because they are afraid they don’t know how they are going to be treated when they go to a STI clinic. They are not aware that they can be treated as good as like other patients are treated. They think maybe they are going to inform the police, they are going to call the police, there may be stigma, and the health workers may be calling each other [to tell them there is a MSM in the clinic]… so they walk around when they have a problem and untreated problem which is bad because it can get complicated. - *Male Participant ID05*

Discrimination is really there most especially when you contract like infections that are acquired through sex, STIs. They even ask you the mode of contraction or who is your partner…Infections which were supposed to be found in the vagina are found in a man from the anus, so the hospital according to their belief, they take you as an abnormal person. So you don’t get the proper treatment because they take it like that they are offering the services to people who are not supposed to get the healthcare service. [*Interviewer:* Does that kind of discrimination lead these men not to get better health care services?]

Yes. It contributes to that because they fear that if they get to such a place they will get discriminated. So they choose not to go to such places. - *Male Participant ID03*

Men reported concern for the layered stigma and blame that could come with HIV infection that may be acquired through same sex practices.

Most of the times they [MSM living with HIV] are taken as people, because of their behaviors, acquired it voluntarily. People regard them as those that are reaping from what they sowed. Their bad behavior has led them to have the effects of the disease. It’s like blaming them, judging them to say that’s what they wanted. - *Male Participant ID04*

Social stigma and criminalization not only influenced fear of unintentional disclosure to others and/or potential arrest among MSM but also led providers to be concerned that there could be political or legal implications associated with providing care to MSM.

They [health providers] have their own fears… Knowing that MSM in Malawi are kind of criminalized and they said that if people know that we are treating them maybe we will be in trouble. But we have sensitized them and we have trained them. So they are familiar with the health problems that MSM face and they have to treat them and handle them and make them comfortable. - *Health Professional ID05*

I personally I think … we are still moving under an umbrella of … not wanting to accept that these people are there. Because of lack of proper legislations to protect these people [MSM], then those are some of the major challenges that are hindering us to be providing the care to those that are providing you know, homosexuality sort of aspects of intercourse. So I think that’s the main area. If we had a way of protecting such type of a group then possibly it could have been very easy for health service providers to take them into consideration. - *Health Professional ID02*

Resource constraints, such as complications with the commodities supply chain, and needs of other populations led providers to additionally feel they do not have the capacity to provide targeted services for MSM.

I think we have got a big problem in terms of resources like condoms. It’s not only condoms I think for me one of the things is …these condoms are distributed free. And sometimes it’s the supply chain; sometimes we don’t have them in the supply chain. So that is the major problem. Once in a while even here we run out of condoms but most of the problems are the supply chain. So I think that affects other peoples. The central hospitals have been affected what more with a peripheral hospital. So I think one of the main problems, it’s like condoms are not available. You know we have this barrier of supply chain." - *Health Professional ID02*

### Cues to action

Cues to action (or lack of) are also important modifying factors in the Health Belief Model for health seeking and providing behaviors for MSM in Malawi. Generally, cues to action can be internal or external (such as information) prompts that encourage health seeking or providing behaviors [37]. Qualitative themes emerged indicating that such messaging to MSM and health practitioners, which could include information about health, anal sex transmission risk, and HIV/STI prevention for MSM, is almost non-existent in Malawi. Instead, prevalent messages were related to HIV risk and prevention among heterosexual and married couples. As a result, MSM report being rarely informed of risk for HIV transmission when having sex with other men and little information on the use of condoms and water-based lubricants during anal intercourse is available. MSM who had received information indicated this came from participation in research investigation or the few available community-based services. The perceptions among participants suggested that negative media attention prevented traditional dissemination of HIV prevention information to MSM.

Because if [same sex practices] can be legalized in such a way, that means also the interventions that might be cropping up, they will be reaching the people engaging in same sex acts…The [current] messages that talk about with HIV AIDS are mainly targeting men/women relationships. The posters that we see in our roads, the adverts that we hear from the radio they only target men/women relationships. This clearly shows those engaging in homosexuality acts, at moment, they [MSM] take it that there is no AIDS amongst us. If they were seeing that we can as well get affected with the AIDS pandemic, then there could have been messages targeting us as well. So, just because we don’t get the messages ignorance is taking charge mostly among us. - *Male Participant ID04*

Likewise, health providers feel they cannot advertise their services to MSM, particularly HIV and STI testing and counseling.

The barrier there is the ways which have already been put in the media… the media has already started discouraging. Now if the discouragement is from the media now on the ground, even if one can say I will lead this, [this person still] cannot even operate an open clinic. We will still even remain in the wrong. To avoid being caught that you are the one encouraging them. - *Health Professional ID03*

### Likelihood of action: perceived benefit minus perceived barriers

The likelihood of MSM to take action and seek health and HIV prevention services are dependent on their perceived benefits weighed against the perceived risks or barriers of seeking care [37]. Few benefits related to health seeking were reported by MSM; most reported little encouragement towards service seeking and several reported that they or their peers often did not seek medical attention for anorectal STIs. In addition, there were significant barriers perceived by both health providers and MSM, which need to be overcome for provision and uptake of health and prevention services by these two populations, respectively.

To the men who have sex with men there seems to be a variety of barriers. I think there are many issues but, mainly, stigma- I think that’s the main issue. Because of that it’s so hostile for them to access the services, it’s so negative. - *Health Professional ID01*

Male participants and some health providers agreed that key barriers related to health seeking among MSM included, but were not limited to, fears and perceptions of stigma in health facility.

I think this can affect healthcare provision because when the more you talk [gossip] much about your patients it’s the more you lose patients. The more you will make the infections spread. So that’s bad because our aim is to make that circle break. But if you start having that attitude bad attitude discriminating them, that means they will not come for treatment and they will remain there and spread that infection and the circle will not end. - *Health Professional ID03*

Maybe some they don’t even know where to get treatment some maybe they have just made themselves to be the outcasts. [The MSM think,] ‘We are not even wanted in the community. So there is no need for us to go, because there are no partners of men who have sex with men who are health workers.’ If there would be health workers who are MSM then they would go direct to them and know who to assist them, in a charming way. - *Health Professional ID03*

Fear of or experienced social isolation following unintentional disclosure of same sex practices was particularly salient. MSM feared breaches of confidentiality in which family members or neighbors would learn of same sex practices through the health providers or other patients.

They [MSM] don’t want to disclose that they are in the group and the other thing is they do not know whom they are going to meet at the clinic. They [MSM] walk in this clinic you find the nurse who is present is your mum’s sister, or she is your mum’s friend, or she is your neighbor…So that puts another fear. - *Male Participant ID05*

Among those MSM who did indicate a desire for HIV testing, some faced the additional barrier related to fear of a positive HIV diagnosis or fear of unintentional disclosure of HIV status to others. A particular concern was status of being both MSM and living with HIV, which was highly stigmatized socially.

It’s still a very difficult situation, he [a MSM living with HIV] will still be a victim of laughing and you can as well say you are a *chinchiman* and you are HIV infected…you don’t have peace of the mind. - *Male Participant ID01*

That is a very big problem because you are a talk of the town, to say you contracted the virus because of your behavior that’s a punishment from GOD like in Sodom and Gomorah. - *Male Participant ID04*

Finally, for service providers, reported likelihood towards providing targeted services for was low. As previously indicated, service providers did not freely feel they could provide services to MSM due to risk of repercussions and being seen enabling or encouraging an illegal practice. Some providers, however, suggested they would provide targeted and comprehensive services if the environment allowed.

I wish we could provide a like a holistic kind of approach. Like family planning, lubricants if we can, general health education like one stop ARTs. So if as I have already indicated we have built a relationship so we would want if [MSM] comes in a room they should get whatever he wants and then he goes back home. The problem is if we have already developed a relationship with him and I refer him to someone else or her to someone else it’s like okay now he doesn’t know where to start from. ‘Should I start from disclosing this?’ And HIV testing and counseling- I think it’s the most important one- with STI treatment and risk reduction counseling. - *Male Participant ID05*

## Discussion

This paper describes the qualitative research that was conducted among MSM and health care providers to understand the low levels of disclosure and high levels of fear related to seeking health services. Access to health information and services, in this case HIV and STI information and services, is key to promoting healthy behaviors [18] and a health right that is often taken for granted. Though individual disclosure of same sex practices to health providers is not always necessary, it is a critical component for risk reduction counseling and ensures that HIV prevention messages can be inclusive of and informative for sexual minority patients. For MSM in Malawi, however, the likelihood of action towards accessing such information through disclosure of risk practices and uptake of services is challenging and influenced by many factors. Similarly, provision of these services to MSM by health professionals is also heavily influenced by perceptions and social contexts.

Through the framework of the Health Belief Model, these results highlight the influential perceptions, modifying factors, and cues to actions related to key health behaviors among MSM and health providers. Most notable among MSM was the complexity of HIV risk and prevention awareness. Participation in research or involvement with community-based organization was the main source of information for those who were knowledgeable of HIV among MSM. However, there was a predominant perception that most MSM in the wider population were unaware of HIV risks or appropriate methods to prevent HIV infection. For many, this knowledge was supplanted by the belief that sexual transmission of HIV only occurs during sex between men and women. While a wealth of HIV prevention messages may exist- billboard displays, media advertisements, public health campaigns- they traditionally focus on the family, particularly transmission between married couples and vertically from mother to child. The absence of information about transmission related to anal sex practices in the context of abundant information related to heterosexual transmission (specifically, vaginal-penile transmission) has, in part, resulted in a population being ill equipped to protect themselves when having sex with other men.

Providers suggested that limited data on the size of and burden of HIV among MSM in Malawi begets a perception among service providers that MSM do not exist in the population or are not at risk for HIV infection. Criminalization and stigmatization of same sex practices limits the collection of these data providing the justification for the continued absence of research and programming for MSM [53,54]. This lack of data has resulted in fewer cues to action or appropriate information that would enable providers to learn about and address the sexual health needs of MSM in a rights-affirming way within health care settings. There are consistent data, however, indicating that MSM do exist in Malawi as they exist in every country [21,55,56]. Awareness of HIV risk and prevention needs of MSM among the general community of providers, as highlighted by this study, remains closely associated with the amount of data available characterizing population size and attributable to the burden of HIV. The science of methods for population size estimations is evolving, including the development of the network scale-up method, and further enhancements to multiplier-based methods. However, there are still significant limitations with the implementation of these methods with hidden and stigmatized populations [57-59]. The highest quality data are derived from settings with limited stigma, and it may be that changes of social norms may need to take place in line with the generation of data to increase cues to action for providers. The low prevalence of adequate cues to action (awareness of risks related to anal sex transmission, existence of MSM within the community, and low knowledge on appropriate prevention and care) suggests that national HIV strategies such as the National HIV Strategy for Malawi must incorporate the multiple forms of education to provide information on both treatment and prevention, as well as reduce damaging social effects related to HIV transmission and acquisition [60].

Criminalization and stigmatization of homosexuality also serve as modifying factors in the provision of, access to, or utilization of services. Whether service providers were or were not individually motivated to provide services for MSM, they commonly reported fear of repercussions related to providing sexual health services or risk reduction counseling to MSM. Similarly, MSM participants were concerned that service providers would submit police reports about their sexual practices. For MSM who present to a health provider with symptoms associated with an anorectal STI, generally pathognomonic of an infection transmitted during anal intercourse, avoiding unintentional disclosure of same sex practices is difficult, if not impossible. It is the fear of inadvertent disclosure of same-sex practices that seems to result in diminished health seeking practices in times of need. Further, these findings suggest that while disclosure of sexual practices is generally encouraged for HIV prevention and sexual health services, there are situations in which such disclosure can be harmful. Because a large percentage of the adult population use public health services, identification of those providers and facilities that are recognized to be non-stigmatizing is a simple method to availing access to services and may increase uptake among MSM. Additional sensitivity training and education on MSM health needs may open other spaces for MSM to access services in the future. Other services or programs specifically targeting MSM may provide alternative points of entry into healthcare. Such MSM-dedicated programs would need to take care to ensure the security of their clients and staff and find additional support where resources are limited.

The fear of seeking services among MSM, however, is not unfounded. During a results validation meeting with the MSM community, one of the participants described a publicized incident in which a service provider reported a patient to the police for homosexual practices when the patient sought treatment of an STI in Lilongwe. Similar scenarios of men concealing STI symptoms and not seeking treatment for anorectal conditions to avoid stigmatization and arrest have been observed in other African settings, such as in Senegal [61]. In this context, the highly publicized incarceration of MSM in 2008 that occurred following the provision of HIV prevention information led to widespread impacts on service provision to and uptake by MSM [62]. More broadly, stigmatization of same sex practices has demonstrated negative impacts on coverage and quality of services for MSM, including HIV prevention campaigns and clinical guidelines that exclude homosexual and bisexual transmission risks, homoprejudiced attitudes in the health setting, and reluctance by MSM to utilize services or disclose sexual risks [26,62,63]. These events demonstrate the influential roles of governments: officials have the power to criminalize same sex practices or, conversely, can promote anti-discrimination laws and equality in other rights, such as marriage. State actions can have a trickle-down effect: political homophobia can fuel social stigma and have damaging effects on HIV prevention, while anti-discrimination laws may allow lesbian, gay men and other MSM, and transgender women to enjoy equality in health and other human rights [64].

Though Malawi’s President Banda recently announced her intention to overturn the ban on homosexuality [65], and the new National HIV and AIDS Strategic Plan calls for structural change and comprehensive services for MSM [66], stigma and homophobia remain prevalent [67]. The Health Belief Model demonstrates how this stigma manifests in limited targeted initiatives and is associated with low risk perception among MSM. Similarly, stigma has led to limited evidence on the HIV risks and needs of MSM by health care providers in the country. Finally, stigma modifies health seeking and health provision practices for MSM in Malawi. While these currently represent barriers to care, they also can be perceived as opportunities in terms of targets for interventions.

With few exceptions, the provision of health care should be confidential. And while MSM and service providers studied here reported that service providers have an obligation to maintain privacy of and serve all patients, both groups of participants studied noted little sense of protection when discussing same-sex practices in the clinic. Even if health-care providers do not support homosexuality themselves, they can be encouraged to offer a supportive and safe environment in which MSM may discuss their behavioral risks and health concerns in order to effectively deliver services to these men to improve health outcomes [18,68]. HIV self-testing, which has been found to be both accurate and acceptable for use among the general adult population in Malawi [69], may be a means for MSM to access private, confidential HIV testing, if provided as an another option for HIV testing and done in a rights affirming manner [70].

Training of physicians and other health providers to ensure culturally and clinically competent care is an important first step in this process. Several resources are available for such health sector training, including those produced by Fenway and MARPs Africa [68,71]. In Kenya, a two-day training using the MARPs Africa module with healthcare workers provided information on MSM and sexual risk, healthcare needs, and HIV prevention. The three-month post-training assessment of this intervention found reductions in homophobic attitudes and improvements in knowledge among trained healthcare workers [72]. Similar training may be effective in Malawi and other low-resource settings. However, the data presented here suggests that these trainings must be coupled with protection and support from policy makers and upper management in health systems, so that they may effectively provide these services without risks to their careers or themselves. This qualitative study in Malawi highlighted many other needs including improved access to condoms and lubricants, mental health services to address depression and other psychosocial events faced by MSM related to coming out and relationships, and access to information about HIV and health risks and methods for prevention for MSM. Encouragingly, many of these needs can be addressed through training and provision of basic HIV prevention commodities.

The most efficacious HIV interventions for MSM revolve around biomedical approaches to decrease transmission and acquisition risks [13]. However, it has been repeatedly stated that these approaches will have limited effectiveness if implemented for hidden populations in stigmatizing or criminalizing contexts. The use of the Health Belief Model as a guiding framework here suggests key targets for behavioral and structural interventions that contextualize the biomedical components of combination HIV prevention programming for MSM in Malawi.

The findings reported here should be viewed in light of several limitations with the research methods. The study was conducted only in the urban setting of Blantyre- the ‘financial capital’ of Malawi, and these findings represent a relatively small, though diverse, sample of MSM participants. While the themes reported here were salient among participants, it is important to recognize that these findings may not represent all of the perceived barriers to the provision and uptake of care for all service providers and by all MSM in Malawi, respectively. Substantial differences, particularly related to experiences and perceptions of stigma and access/uptake of HIV and sexual health services, may exist across urban and rural settings. Sample sizes were low due to challenges of recruiting this population; however, the redundancy of themes heard across interviews with healthcare workers and MSM suggest proximity to saturation. A second limitation is related to the use of the Health Belief Model; this model is often used as model for quantitative intervention research. Because this study was qualitative, the Health Belief Model provides a framework for understanding provision and access to services but quantitative, causal associations cannot be made. These data, however, draw strength from providing novel insight into the factors that are perceived to be salient in suboptimal access and utilization of health and HIV services for MSM in Malawi from the perspective of both the provider and end-user of these health services.

## Conclusions

These findings contribute to an overall understanding of barriers and perceptions among MSM and providers that should be addressed in the course of intervention planning. The results highlight the need for appropriate information and messaging on HIV risks among MSM; health sector interventions to increase cultural and clinical competency to address issues related to MSM among service providers; and the need to increase the safety of uptake and provision of these services for MSM.

## Competing interests

The authors declare that they have no competing interests.

## Authors’ contributions

SB, CB, GT, AW, and EU collaborated in the design and oversight of the study. AW and SB, developed interview guides; VJ and DK conducted in-depth interviews; and AW and RG conducted qualitative coding and analysis. AW and SB interpreted the results. AW wrote the initial drafts of this manuscript and co-authors reviewed and contributed additional inputs. All authors had full access to the data, reviewed and edited the manuscript, and all take responsibility for its integrity as well as the accuracy of the analysis. All authors read and approved the final manuscript.

## Pre-publication history

The pre-publication history for this paper can be accessed here:

http://www.biomedcentral.com/1472-698X/14/20/prepub
